# Perioperative care of a child with non-ketotic hyperglycinemia

**DOI:** 10.4103/1658-354X.71578

**Published:** 2010

**Authors:** Joy Allee, Joseph D. Tobias

**Affiliations:** *Department of Anesthesiology, University of Missouri, Columbia, Missouri*; 1 *Departments of Anesthesiology & Pediatrics, Nationwide Children’s Hospital and The Ohio State University, Columbus, Ohio*

**Keywords:** *Non-ketotic hyperglyceinemia*, *tonsillectomy*, *obstructive sleep apnea*

## Abstract

Non-ketotic hyperglycinemia (NKGH) is an autosomal recessive disorder of glycine metabolism. Defective glycine cleavage results in elevated concentrations of glycine in plasma, urine and cerebrospinal fluid. The accumulation of glycine, an inhibitory neurotransmitter, leads to a clinical presentation of apnea, lethargy, hypotonia, seizures, and severe psychomotor retardation. There are four clinical variants of NKHG, which have been described in the medical literature. Neonatal NKHG is the most common as well as the most devastating and lethal form of the disorder. Given the multi-system involvement of the disorder, there are several perioperative concerns of such patients with delayed emergence requiring supported ventilation being a common postoperative outcome for NKHG patients. We report the perioperative management of a 4-year-old boy with NKGH who required anesthetic care during an adenoidectomy and tonsillectomy for obstructive sleep apnea.

## INTRODUCTION

Glycine encephalopathy, also known as non-ketotic hyperglycinemia (NKHG), is an autosomal recessive disorder caused by a defect in the glycine cleavage system. NKHG is classically associated with neonatal apnea, lethargy, hypotonia, and seizures followed by severe psychomotor retardation. Glycine, like gamma amino-butyric acid (GABA), is an inhibitory neurotransmitter in the central nervous system (CNS). However, both inhibitory and excitatory CNS features may occur in NKHG. To date, there are no reports regarding the perioperative care of such patients. We present a 4-year-old boy with NKHG who required adenoidectomy and tonsillectomy for obstructive sleep apnea (OSA). The perioperative care of such patients is discussed.

## CASE REPORT

Review of this patient’s medical records and presentation of this case report was conducted within the standards of the Institutional Review Board of the University of Missouri. The patient was a 4-year-old, 12.7 kg boy who presented for a tonsillectomy and adenoidectomy for OSA. Prior to the scheduled surgery, he was evaluated in the ENT clinic for suspected upper airway obstruction and chronic aspiration, and was determined to have OSA documented by a sleep study. His past medical history was significant for NKHG, developmental delays, static encephalopathy, and multiple admissions for treatment of a partial complex seizure disorder and other medical disorders related to NKHG. His past surgical and procedural history included a recent Nissen fundoplication, G-tube placement, and serial injections of botulinum toxin for treatment of spasticity. Following the recent Nissen fundoplication, he required postoperative mechanical ventilation due to prolonged awakening. Current medications, all administered via his G-tube, included sodium benzoate 300 mg TID, phenobarbital 40 mg BID, loratadine 2.5 mg every day, levocarnitine 300 mg BID, vitamin B6 (pyridoxine) 25 mg once a day, levetiracetam 300 mg BID, leucovorin 15 mg every morning, glycopyrrolate 1 mg TID, baclofen 20 mg BID, dextromethorphan 60 mg TID. The patient had no known drug allergies. Physical examination revealed a neurologically impaired child, who was awake and alert, but non-communicative. He was microcephalic. There were random eye and head movements. The examination of the oral cavity and oropharynx was remarkable for marked tonsillar hypertrophy. Heart and lung sounds were normal. There were no signs of stridor, retractions, or cyanosis. His muscle tone was increased in all extremities. Vitals included a temperature of 37.4°C, pulse rate of 102 beats/minute, respiratory rate of 24 breaths/minute, blood pressure of 130/66 mmHg, and an oxygen saturation of 96% on room air. Laboratory data revealed a white blood cell count of 19,700/mm^3^, hemoglobin of 15.4 gm/dL, hematocrit of 43%, and a platelet count of 429,000/mm ^3^. During the sleep study, the lowest O_2_ saturation recorded was 86%, which occurred with a 22 s partial episode of OSA. End-tidal CO _2_ waveforms were suboptimal due to mouth breathing. There were no central apneas and no episodes of periodic breathing. There were 33 obstructive apneas and 39 partial obstructive apneas. The apnea-hypopnea index was 10.7.

The patient was held *nil per os* for solids for 6 h and clear liquids for 2 h. No premedication was administered and he was transported to the operating room where standard ASA monitors were placed. Prior to the administration of any medications, a bispectral index (BIS) monitor was placed and demonstrated an awake BIS value of 41. The patient was preoxygenated with 100% oxygen and a 22 gauge peripheral intravenous catheter was placed. Anesthesia was induced with remifentanil (2 μg/kg) and propofol (2.5 mg/kg). After demonstration of adequate bag-valve-mask ventilation, endotracheal intubation was facilitated by cis-atracurium (0.15 mg/kg). Additional medications included dexamethasone (0.5 mg/kg), glycopyrrolate (5 μg/kg), ondansetron (0.15 mg/kg), and acetaminophen (40 mg/kg per rectum). Using train-of-four (TOF) monitoring, the ablation of the T _4_ occurred at 80 s. Anesthesia was maintained with 70% nitrous oxide in oxygen and a remifentanil infusion was started at 0.2 μg/kg/min. During the procedure, the BIS varied from 32 to 55. Remifentanil was titrated according to the hemodynamic parameters in a dose that varied from 0.2 to 1 μg/kg/min. No additional *cis*-atracurium was administered. The return of T_1_ of the TOF was noted at 15-17 min and four twitches were present at 20-22 min. The surgical procedure was completed in 25-30 min and residual neuromuscular blockade was reversed with neostigmine and glycopyrrolate. The remifentanil infusion was discontinued and within 10 min, the patient’s trachea was extubated. Postoperatively while in the post-anesthesia care unit, he received two doses of nalbuphine (total dose of 0.1 mg) for analgesia. With both of these doses of nalbuphine, the patient’s respiratory rate decreased to 8-10 breaths per minute and his agitation ceased. He was admitted to the Pediatric ICU for monitoring. On postoperative day #1, he developed an oxygen requirement and became febrile. A postoperative chest radiograph was unremarkable. Over the next 24 h, he required frequent suctioning of the oropharynx to help with the clearance of secretions. The patient’s temperature returned to normal and his respiratory status improved. On postoperative day #2, he was discharged home.

## DISCUSSION

NKHG, also termed glycine encephalopathy, is an autosomal recessive disorder of glycine metabolism. This rare, but severe neurologically disabling disorder, has an incidence of approximately 1:200,000. A defect in the mitochondrial glycine cleavage system results in an elevation of the glycine concentration in the plasma, urine and cerebrospinal fluid (CSF).[1] The mitochondrial glycine enzyme complex is made up of four proteins, which are encoded in four different chromosomes. These mitochondrial proteins are designated P (pyridoxal phosphate containing), H (lipoic acid containing), T (tetrahydrofolate requiring), and L (lipoamide dehydrogenase). Mutations in the P-protein complex account for more than 80% of the cases of NKHG, while a mutation of the T-protein system is the second most common defect accounting for approximately 15% of the cases. Severe deficiencies of any of the enzyme systems, as demonstrated in our patient, result in loss of enzyme activity, the accumulation of glycine, and severe neurologic sequelae. Deficient, but residual enzyme activity accounts for mild phenotypes, which may present later in life (see below).[2,3] Like γ-amino-butyric acid (GABA), glycine serves as an inhibitory neurotransmitter in the CNS, especially in the spinal cord, brainstem, and retina. When glycine receptors are activated, chloride enters the neuron via ionotropic channels resulting in an inhibitory postsynaptic potential. Glycine and glutamate are also required co-agonists for the N-methyl-d-aspartate (NMDA) receptor system. In contrast to the inhibitory role of glycine in the spinal cord, excessive activation of the NMDA receptor by glycine can lead to excitotoxicity of neurons in the cerebral cortex, hippocampus, and cerebellum. Excessive activation of this system can result in cell death. These neurotransmitter effects of glycine are thought to be responsible for the clinical features of NKHG.[4,5] Classical presenting signs and symptoms of NKHG include apnea, lethargy, hypotonia, intractable hiccups, and refractory seizures early in the neonatal period. These neurologic manifestations, if unrecognized and untreated, can progress to coma and death.[6]

There have been four clinical variants of NKHG described in the medical literature. Neonatal NKHG is the most common as well as the most devastating and lethal form of the disorder. As noted in our patient, it generally presents in first few days of life with poor feeding, failure to suck, lethargy, hypotonia, apnea, coma, and seizures. Even with supportive therapy, 30% of infants die and the remainder develop profound psychomotor retardation and intractable seizures. Infantile NKHG presents in a similar manner as the neonatal form; however, signs and symptoms develop after 6 months of age in a previously healthy infant with seizures being the most common presenting sign. The infantile form generally has a milder course than the neonatal form although there is some degree of developmental delay. Late onset NKHG has only been described in a few patients with the age of onset varying from 2 years into the third decade of life. The transient form of NKHG is also exceedingly uncommon. It presents in a manner similar to that of the neonatal form, but there is normalization of the blood and CSF glycine levels and resolution of the signs and symptoms by 2 to 8 weeks of life.[6]

The laboratory diagnosis of NKHG begins with the measurement of plasma and CSF glycine levels. A CSF/plasma glycine ratio ≥ 0.08 is considered diagnostic for NKHG. Confirmation of the diagnosis requires mitochondrial enzymatic analysis obtained from a liver biopsy, which is typically not done in most patients because of the difficulty in obtaining an adequate sample size. Early treatment with sodium benzoate and dextromethorphan has been shown to improve clinical and electrophysiologic outcome in some cases. Sodium benzoate, after activation to its coenzyme A ester, conjugates with glycine to form hippurate, which is efficiently excreted in the urine. Providing high doses of benzoate allows for the removal of large amounts of glycine in urine and results in a reduction of plasma glycine levels to normal. This treatment reduces seizures and improves alertness, but does not prevent the development of mental retardation. Additional therapy which may be helpful in the control of seizures and other neurologic manifestations of NKHG includes dextromethorphan. Dextromethorphan antagonizes the effects of glycine at the NMDA receptors, thereby reducing the seizure frequency and improving electroencephalographic findings in NKHG patients.[7,8]

The challenges regarding the anesthetic management of patients with NKHG are related to the potential interaction of various anesthetic agents and the neuroinhibitory pathophysiology of the disorder as well as the various end-organ manifestations of the disordered. Delayed emergence from general anesthesia was noted previously in our patient following the use of sevoflurane and fentanyl during anesthetic care for a Nissen fundoplication. Liu and Fan reported delayed emergence from anesthesia following the administration of only sevoflurane during performance of a bronchoscopy in a 3-year-old girl with NKHG.[9] The patient failed to awaken at the completion of the procedure when the sevoflurane was discontinued. Forty-five minutes later, the end-tidal gas monitor showed no residual sevoflurane and the patient began to have slow, but weak spontaneous ventilation. No response was noted following the administration of aminophylline (5 mg/kg). Ventilation was assisted by mask for an additional hour and the patient was then transferred to the post-anesthesia care unit.

Our patient presented with an awake BIS value of 41. Therefore, we attempted to tailor our anesthetic care to provide a rapid awakening by avoiding the use of the potent inhalational anesthetic agents and instead using a technique employing nitrous oxide and the short acting opioid remifentanil. Given its metabolism by non-specific esterases, there are limited changes in its pharmacokinetics regardless of the patient’s age or the presence of co-morbid disease processes.[10,11] Even in the neonatal population, the pharmacokinetics of remifentanil are unchanged thereby resulting in limited effects on postoperative respiratory function. In our patient, remifentanil was titrated in a dose varying from 0.2 to 1 μg/kg/min to maintain the hemodynamic parameters close to baseline. Even with the use of remifentanil doses up to 1 μg/kg/min, upon discontinuation, there was the prompt return of ventilatory function and the patient’s trachea was extubated within 10 min of completion of the surgical procedure. Although an argument could be made for the use of the short-acting inhalational anesthetic agents such as desflurane and sevoflurane, sevoflurane was used during the performance of the Nissen fundoplication and may have contributed to the prolonged awakening, perhaps due to its higher fat solubility when compared to nitrous oxide or desflurane. Therefore, we chose to use propofol for induction and nitrous oxide and remifentanil for maintenance anesthesia.

An additional concern in our patient was postoperative respiratory function as he presented with signs and symptoms of OSA due to tonsillar hypertrophy. The potential for airway compromise was augmented by other co-morbid factors including hypotonia, frequent seizures, and the baseline altered mental status (awake BIS of 41). These factors may have been further magnified by the potential for pharyngeal edema related to the surgical procedure. In such patients, the use of short-acting anesthetic agents may also be beneficial as it would help to eliminate the effects of residual anesthetic agents on respiratory function and the control of upper airway patency. The potential for postoperative respiratory compromise in patients with co-morbid diseases such as NKHG is illustrated by our patient who developed an oxygen requirement and became febrile on postoperative day 1. Although a postoperative chest radiograph was unremarkable, these clinical signs and symptoms were likely related to atelectasis further emphasizing the need for close postoperative monitoring of respiratory function in such patients. Additionally, during the initial 24 h postoperative period, frequent suctioning was required to facilitate the clearance of secretions. We would also suggest that given the neurologic involvement that is present with NKHG, there may be the potential for increasing sensitivity to opioids in patient as there was a rather dramatic decrease in our patient’s respiratory rate when nalbuphine (0.1 mg) was administered for the treatment of pain in the post-anesthesia care unit.

An additional concern in any patient with hypotonia is the choice of neuromuscular blocking agent (NMBA). Although an NMBA was not necessary for the completion of the surgical procedure in our patient, given that we were using a nitrous oxide-opioid based technique, we chose to use a single dose of NMBA to facilitate endotracheal intubation. Although there is no information regarding the use of neuromuscular blocking agents in patients with NKHG, we chose to extrapolate data from other patients with hypotonia and therefore would caution against the use of succinycholine and suggest that a short-acting non-depolarizing agent may be most appropriate. In patients with various myopathic conditions, previous authors have reported the successful use of mivacurium, which unfortunately is no longer available.[12,13] As such, we chose to use cis-atracurium given its non-organ-dependent elimination and its short duration of action. The use of cis-atracurium proved to be safe and effective while maintaining a normal recovery profile in our patient. Following a dose of 0.15 mg/kg, ablation of T_4_ of the TOF occurred at 80 s. T_1_ of the TOF was noted at 15-17 min and four twitches were present at 20-22 min. Despite the spontaneous return of neuromuscular function, we chose to reverse residual neuromuscular blockade to eliminate any potential for residual weakness which might affect postoperative respiratory function. Alternatively, if avoidance of a NMBA was desired, successful endotracheal intubation can be accomplished under deep sevoflurane anesthesia or with a combination of propofol and remifentanil.[14,15]

A universal finding in patients with NKHG is the presence of seizures. In general, most patients with seizures require no special perioperative management other than that of the underlying disease. We would recommend the documentation of adequate preoperative plasma concentrations of anticonvulsant medications and the continuation of perioperative dosing. This may include preoperative administration on the morning of surgery and intraoperative dosing as required. Intravenous preparations of many anticonvulsant medications are available and these should be administered intraoperatively to maintain therapeutic serum concentrations and avoid perioperative seizures which may impact on postoperative respiratory function. Postoperatively, if intravenous preparations are not available, rectal administration may be feasible for many of these medications. In patients with NKHG, other medications are administered to lower plasma glycine concentrations (sodium benzoate) or antagonize the effects of glycine (dextromethorphan). Ongoing administration of these medications should also be continued perioperatively including preoperative dosing on the morning of surgery.

Given the involvement of the NMDA system in the pathogenesis of the disease process, it has been suggested that other medications which block the NMDA receptor such as ketamine or magnesium should be considered during anesthetic care in patients with NKHG.[16,17] Given its potential excitatory effects on the CNS and longer plasma half-life when compared with propofol, we chose not use to ketamine. Given that our patient was being treated with sodium benzoate and dextromethorphan, we did not think that additional agents acting at the NMDA receptor were necessary during this brief procedure. Additionally, magnesium’s effects at the neuromuscular junction include decreased release of acetylcholine with side-effects including vasodilatation resulting in hypotension or residual muscle weakness. Given the frequent occurrence of hypotonia in patients with NKHG, we would caution against the use of magnesium.

Like many other neurological disorders that result in altered mental status and hypotonia, NKGH patients may be prone to gastroesophageal reflux and thereby at risk for perioperative acid aspiration and its consequences. In our patient, we felt that this risk had been minimized by the previous performance of a Nissen fundoplication and lack of parental reports of issues related to gastroesophageal reflux such as vomiting. If there are concerns regarding the potential for acid aspiration, typical therapies to be considered include rapid sequence induction, the application of cricoid pressure, or the administration of medications with either decrease the pH of gastric secretions (non particulate antacids, proton pump inhibitors or H_2_ -antagonists) or agents which speed gastric motility and emptying (metoclopramide) thereby decrease the volume of gastric secretions.

In conclusion, we suggest that the perioperative management of patients with NKHG should involve tailoring the anesthetic care to enable rapid awakening and avoidance of residual anesthetic effects which may impact postoperative respiratory function and upper airway control. When necessary, the choice of NMBA should consider the myopathic nature of the disorder. Additional perioperative concerns include ongoing administration of medications aimed to control plasma glycine levels and its end-organ effects as well as continuation of anticonvulsant medications. Given the progressive CNS deterioration that accompanies this disorder, aspiration prophylaxis should be considered in patients with gastroesophageal reflux. Postoperative monitoring should be considered given the potential for perioperative airway and respiratory complications.
